# Enhanced Corrosion Resistance of OL 37 Steel in Hydrochloric Acid Using a Novel Composite Polymer Film

**DOI:** 10.3390/ma18235351

**Published:** 2025-11-27

**Authors:** Florina Branzoi, Elena Ionela Neacsu, Marius Alexandru Mihai, Alexandru Paraschiv

**Affiliations:** 1 Institute of Physical Chemistry-Ilie Murgulescu, 202 Splaiul Independenţei, 060021 Bucharest, Romania; 2 Institutul Național de Cercetare-Dezvoltare Turbomotoare COMOTI (INCDT COMOTI), 220D Iuliu Maniu Av., Sect 6, 061126 Bucharest, Romania

**Keywords:** polymers, composite coating, electrodeposition, corrosion protection, carbon steel, electrochemical characterization, SEM, FT-IR

## Abstract

This study investigated the electrochemical deposition of a novel composite polymer, 3-methylpyrrole–sodium dioctyl sulfosuccinate/3-methylthiophene (P3MPY–AOT/P3MTP), as protective coatings on OL 37 steel for anticorrosion applications. The anionic surfactant sodium dioctyl sulfosuccinate used in the deposition process enhances the protective efficiency of the coating. The coating films were characterized by CV (cyclic voltammetry), FT-IR (Fourier-transform infrared spectroscopy), and SEM (scanning electron microscopy). The anticorrosive properties of OL 37 steel coated with P3MPY-AOT/P3MTP were investigated in 1 M HCl using potentiostatic and potentiodynamic polarization, as well as electrochemical impedance spectroscopy (EIS). The coated samples exhibited a corrosion rate nearly ninefold lower than the bare substrate, with protection efficiencies exceeding 90%. Optimal performance was obtained for electrochemical deposition of P3MPY-AOT/P3MTP at potentials of 1.0, 1.2, and 1.4 V, current densities of 3 and 5 mA/cm^2^, and a molar ratio of 5:3 for 20 min. The influence of electrochemical polymerization parameters—including applied potential, current density, scan rate, cycle number, and monomer ratio—on the anticorrosion efficiency of the coatings was systematically evaluated, allowing the identification of optimal synthesis conditions. Overall, the results confirm that P3MPY-AOT/P3MTP coatings provide highly effective corrosion protection for OL 37 steel in acidic environments.

## 1. Introduction

Through all metals, iron and its alloys are widely used in various industries such as engineering, construction and automotive manufacturing, owing to their outstanding functional properties and relatively low cost. Nevertheless, their main limitation is their susceptibility to corrosion. The corrosion of metals constitutes a major economic burden, incurring substantial annual costs, of which only a limited proportion is currently alleviated through existing prevention measures. Metallic corrosion causes damage that extends beyond economic losses, contributing significantly to environmental pollution and raising global concern regarding its broader implications. The implementation of advanced corrosion prevention technologies is therefore essential to effectively mitigate these impacts [1,2,3,4]. In recent years, the application of conductive polymers as advanced functional coatings has emerged as a promising approach in surface engineering for the protection of metallic substrates [5,6,7,8]. Considerable progress has been made in the development of polymer-based coatings on metals and their alloys, establishing them as an important strategy in modern anticorrosion technologies. Protective coatings, including composite polymer coatings, corrosion inhibitor films, self-assembled monolayers, graphene-based films, and molecular sieve coatings, remain central to corrosion mitigation strategies [9,10,11,12,13,14]. When applied in corrosive environments, these coatings provide efficient protection through physical barrier formation, adsorption-induced surface passivation, and suppression of charge-transfer processes, making them particularly advantageous under acidic conditions [15,16,17,18,19,20]. The development of novel composites through the incorporation of different monomers has been undertaken to tailor the physicochemical properties of protective coatings, with the aim of providing long-term barrier performance, improving interfacial adhesion, and enhancing electrochemical behavior [21,22,23,24]. These advanced composite coatings have been shown to significantly improve the corrosion resistance of metals when exposed to aggressive environments. The protective action of conductive polymers has been attributed to multiple mechanisms. They are capable of creating an electric field on the metal surface, thereby reducing electron transfer from the substrate to the oxidizing environment [25,26,27,28]. Simultaneously, these polymers form a compact, low-porosity layer that serves as a physical barrier between the metal and aggressive species. Additionally, they promote the formation of a stable and adherent metal oxide layer on the metal surface, providing synergistic barrier and electrochemical protection. Such multifunctional behavior has made conductive polymer coatings a promising strategy in modern corrosion mitigation approaches. Conductive polymers, including polypyrrole, polythiophene, polyaniline and their derivatives, exhibit a unique combination of physicochemical, electrochemical, and optical properties, making them highly promising for applications as protective surface films, chemical and biological detection systems, advanced energy storage units, and organic solar energy conversion devices [11,29,30,31]. The multifunctionality of these materials has spurred extensive research and development, highlighting their potential as versatile components in advanced functional applications. One of the primary challenges in employing conductive polymers for metal anticorrosion inhibition is their inherent permeability to water, which can facilitate the migration of aggresive species to the metal substrate. Polypyrrole derivatives demonstrate superior protective performance compared to pyrrole, largely due to the hydrophobic character introduced by the methyl group [32,33,34]. These polymers are particularly attractive because they combine environmental stability with intrinsic conductivity, stemming from their heterocyclic monomeric units, which structurally and functionally resemble conventional corrosion inhibitors [9,35,36]. Consequently, polypyrrole, polythiophene and its derivatives are considered promising candidates for long-term anticorrosion applications, offering both barrier and electrochemical protection [36,37,38]. Conductive polymer coatings have been applied in a variety of contexts where steel components require enhanced corrosion resistance. For instance, materials such as polyaniline and polypyrrole have been used to protect carbon steel pipelines exposed to moist or saline soils, as well as structural elements in marine and offshore environments, where active passivation provided by these coatings significantly reduces degradation in seawater. Similar coatings have been employed on steel tanks and process vessels in the chemical and food industries, where maintaining a stable, passive surface is essential for operational safety and product purity [39,40,41]. In civil engineering, conductive polymers have also been used to delay chloride-induced corrosion of steel reinforcement in concrete, either as a direct protective layer or as a primer prior to embedding. Additional applications include steel components in automotive systems, industrial machinery subjected to aggressive media, and hybrid protective–sensor systems in which the conductive polymer layer offers both anticorrosive protection and functional electrical properties [42,43]. This study focuses on the development of a novel composite material for the anticorrosion protection of metallic substrates, coupled with the optimization of electrodeposition techniques to produce dense, homogeneous, and strongly adherent coatings. The coatings are further tailored to achieve enhanced protective performance, providing both barrier and electrochemical resistance in aggressive environments, and represent a promising strategy for advanced multifunctional protective coatings in demanding industrial applications.

The scope of this study is to investigate the protective behavior against corrosion of novel P3MPY–AOT/P3MTP composite films in acidic media. The coatings were electrodeposited on OL 37 electrodes using potentiostatic and galvanostatic techniques from a solution containing 0.1 M 3-methylpyrrole, 0.1 M 3-methylthiophene, and 0.03 M sodium dioctyl sulfosuccinate in 0.3 M oxalic acid. The composite materials were characterized by FT-IR spectroscopy, cyclic voltammetry, and scanning electron microscopy (SEM)- energy-dispersive X-ray spectroscopy (EDS). Corrosion behavior of OL 37 coated with P3MPY–AOT/P3MTP was evaluated using potentiodynamic polarization and electrochemical impedance spectroscopy (EIS) in 1 M HCl. The results demonstrated that these composite coatings provide excellent protective performance for OL 37 in acidic conditions. This study extends previous work aimed at developing and evaluating effective composite coating films for corrosion protection for metal-based materials in aggressive environments.

## 2. Experimental

### 2.1. Materials and Methods

OL 37 steel was employed as working electrodes, with the following composition: C 0.15%, Si 0.09%, Mn 0.40%, Fe 99.293%, P 0.023%, S 0.020%, Al 0.022%, Ni 0.001%, Cr 0.001%. The aggressive medium was 1 M HCl prepared by diluting AG 37% HCl (Merck) with bidistilled water. Reagent-grade 3-methylpyrrole (3MPY), 3-methylthiophene (MTP), sodium dioctyl sulfosuccinate (AOT), and H_2_C_2_O_4_·2H_2_O were obtained from Aldrich (>98%). Bidistilled water was used for all solutions. Poly-(3MPY–AOT/3MTP) was electrosynthesized on OL 37 steel in 0.3 M oxalic acid (H_2_C_2_O_4_) containing 0.1 M 3-methylpyrrole, 0.1 M 3-methylthiophene, and 0.03 M sodium dioctyl sulfosuccinate. A PGZ 301 potentiostat (Voltamaster 4 software), Radiometer analytical, Paris, France was used for all electrochemical experiments, employing a cylindrical glass cell with a three-electrode configuration, using OL 37 steel (0.5 cm^2^) as the working electrode, platinum as the counter electrode, and a saturated calomel electrode (SCE) as the reference.

**Scheme 1:**
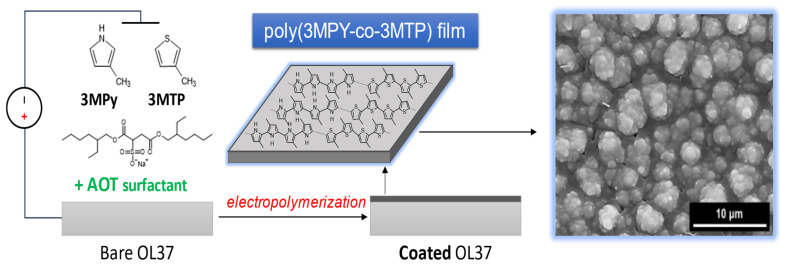
Illustration of the process for depositing the composite layer.

Before each test, the working electrode was polished with emery paper (600–4000 grit), ultrasonically cleaned in acetone, rinsed with ultrapure water, and dried at room temperature. Prior to electropolymerization, the OL 37 surface was pretreated in 0.3 M oxalic acid solution by cyclic voltammetry, between −0.5 and +1.6 V, at a scan rate of 20 mV/s for three cycles. Poly(3-methylpyrrole–AOT/3-methylthiophene) (P3MPY–AOT/P3MTP) coatings were electrodeposited onto the passivated OL 37 surface from a solution containing 0.1 M 3-methylpyrrole, 0.03 M AOT, 0.1 M 3-methylthiophene, and 0.3 M oxalic acid, using both potentiostatic and galvanostatic methods (Scheme 1). Electropolymerization was performed potentiostatically at applied potentials of 1.0, 1.2, and 1.4 V, and galvanostatically at current densities of 3 and 5 mA/cm^2^. Experiments were conducted at distinct monomer molar ratios (5:3 and 3:5) for deposition times of 10- and 20-min. Cyclic voltammetry in 0.3 M H_2_C_2_O_4_ was used to study the electrochemical characteristics of the composite coating polymers. The adhesion of the composite film was assessed using an advanced version of the “standard tape test”, in accordance with ASTM D3359–Method B (cross-cut).

**Scheme 2:**
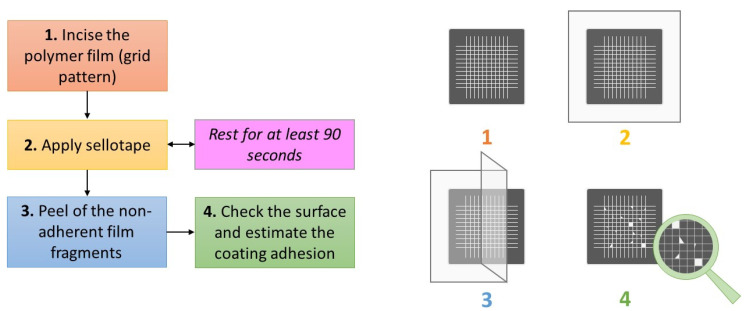
Illustration of the coating adhesion assessment using the standard tape test.

In this procedure, a grid of equidistant cuts is made on the film surface, adhesive tape is applied over the cross-hatched area, pressed to ensure full contact, and subsequently removed. Adhesion is quantified by calculating the ratio of undamaged squares to the total number of squares within the grid (Scheme 2). The protective corrosion of the composite layer and uncoated OL 37 sample was evaluated using potentiodynamic polarization techniques, as well as electrochemical impedance spectroscopy (EIS) in hydrochloric acid. Tafel polarization curves were obtained by sweeping the potential from the cathodic to the anodic region relative to the open-circuit potential (OCP), with all potentials measured against the reference electrode. To ensure reliability, each experiment was repeated at least three times, enabling the results to be averaged while reducing the impact of random errors or fluctuations. EIS measurements were performed at the OCP of the sample–solution interface, over a frequency range from 100,000 Hz to 0.01 Hz. An AC signal of 10 mV amplitude was used and ten data points were collected per logarithmic decade.

### 2.2. Instruments

**Scheme 3:**
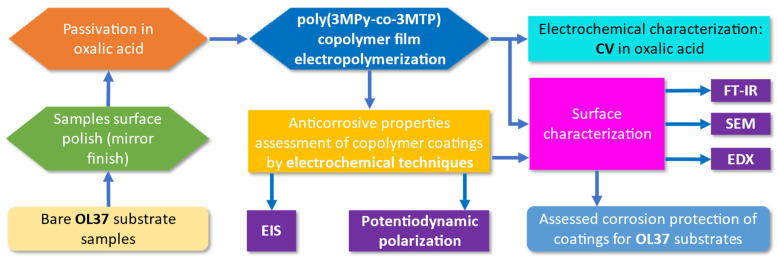
Illustration of the electrodeposition and characterization of the P3MPY–AOT/P3MTP composite films onto the OL 37 substrate.

A VoltaLab PGZ 402 potentiostat/galvanostat ((Radiometer analytical, Paris, France) was used for all electrochemical measurements. Composite coatings were characterized using a Bruker Optics FT-IR spectrometer (ATR mode), Ettlingen, Germany, in the spectral range 4000–650 cm^−1^, with a resolution of 4 cm^−1^. The surface morphology of the coated samples was examined by scanning electron microscopy (SEM). Film evolution was examined via morphological and microcompositional analyses using an FEI Inspect F50 SEM (FEI Company, Brno, Czech Republic) coupled with an EDAX APEX 2i EDS system with an Apollo X SDD detector (EDAX Inc., Ametek, Mahwah, NJ, USA). To minimize charging effects and improve image quality, the samples were sputter-coated with a thin gold layer using an SC7620 Mini Sputter Coater/Glow Discharge System (Quorum Technologies, Laughton, East Sussex, UK). SEM micrographs were recorded at magnifications of ×1000, ×10,000, and ×20,000, using an accelerating voltage of 15 kV, a working distance of 10–11 mm, a spot size of 3.0, and a dwell time of 30 µs. EDS spectra were acquired to identify and quantify surface elements, with up to 1200 counts targeted to ensure a suitable signal-to-noise ratio and reliable element detection. Scheme 3 presents the synthesis and characterization procedure of the polymeric composite electrodeposited on the OL 37 electrode surface.

## 3. Results and Discussion

### 3.1. Electrodeposition of P3MPY–AOT/P3MTP/OL 37

**Figure 1:**
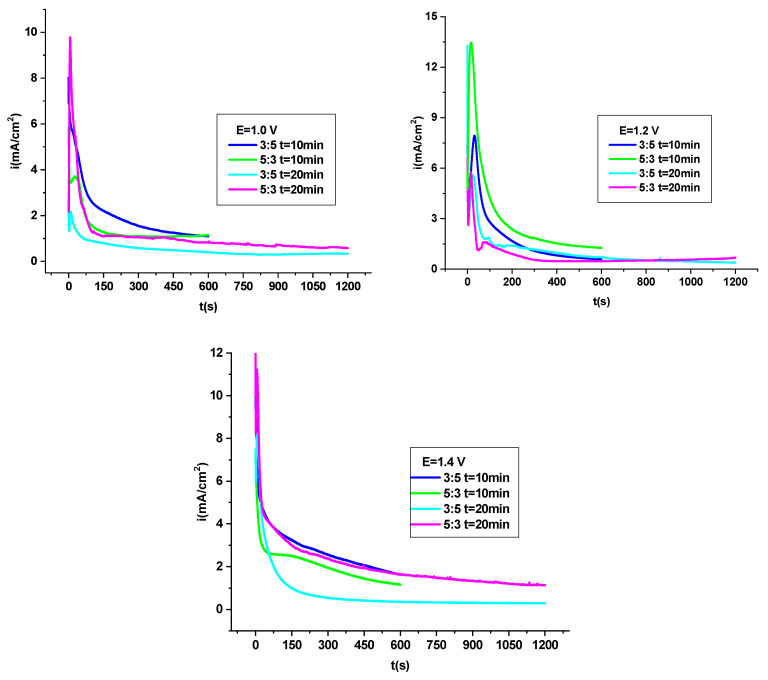
Potentiostatic electrodepositions of P3MPY–AOT/P3MTP/OL 37 carried out at 1.0, 1.2, and 1.4 V versus SCE for 600–1200 s, illustrating the influence of different molar ratios on the deposition process.

From Figure 1, the current density vs. time curves corresponding to the electrodeposition of P3MPY–AOT/P3MTP/OL 37 coating films at applied potentials of 1.0, 1.2, and 1.4 V versus SCE under various molar ratios can be observed. After 600 and 1200 s of oxidation, the characteristic shape of the current density vs. time curves indicates that the deposition process proceeds via a nucleation and growth mechanism on the OL 37 substrate. At the initial stage, the current density decreases sharply, which can be attributed to the electro-adsorption of H_2_C_2_O_4_ and 3MPY–MTP monomers onto the electrode surface. As the applied potential increased from 1.0 V to 1.4 V, a noticeable rise in the initial current density was observed, indicating an enhanced oxidation rate and a higher degree of monomer activation at the electrode surface. This behavior suggests that the electrodeposition kinetics are strongly potential-dependent, promoting a faster nucleation process and a more compact polymer layer formation at higher potentials [8,26,27]. Furthermore, variations in the molar ratio of the P3MPY–AOT/P3MTP system affected both the current density profile and the overall film growth, reflecting differences in polymerization efficiency and interactions between the monomers and the surfactant species within the electrochemical medium.

After approximately 30 s, the current density began to increase, which can be attributed to the dissolution of the passive layer and the subsequent growth of the polymer on the OL 37 surface. Toward the end of the electrochemical oxidation process, the current stabilized, indicating that a uniform and adherent polymer layer had been successfully formed on the OL 37 substrate.

Graph 2 presents the “potential–time” curves recorded during the electrodeposition of the polymer film composed of poly (3-methylpyrrole–sodium dioctyl sulfosuccinate/3-methylthiophene) on the OL 37 substrate at various current densities and molar ratios. During the electrochemical oxidation times of 600 s and 1200 s, the shape of the potential–time curves indicates that the composite coating formed on the OL 37 substrate through a nucleation and growth process [8,35,36].

**Figure 2:**
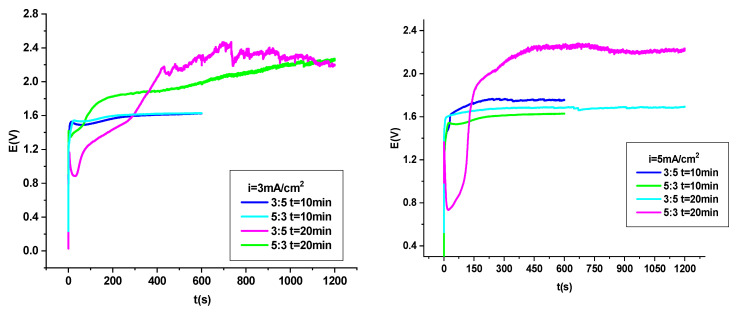
Galvanostatic electrodeposition of P3MPY–AOT/P3MTP/OL 37 obtained at current densities of 3 and 5 mA·cm^−2^ versus SCE for deposition times between 600 and 1200 s under various molar ratios.

As shown in Figure 2, at current densities of 3 mA/cm^2^ and 5 mA/cm^2^ and specific molar ratios, a short induction period—less than 10 s—was observed during the deposition of the 3MPY-AOT:3MT composite. The induction time decreases with increasing molar ratio, which is accompanied by a rise in the nucleation potential. The electropolymerization potential varies between 1.6 V, 1.2 V, and 2.2 V versus SCE for current densities of 3 mA/cm^2^ and 5 mA/cm^2^, corresponding to molar ratios of 5:3 and 3:5 for 3MPY-AOT and 3MT, respectively. At a current density of 5 mA/cm^2^, the coating films demonstrated the most homogeneous and well-adherent morphology. Visual inspection of the OL 37 substrate revealed a uniform black layer corresponding to the P3MPY–AOT/P3MTP coating, indicating a compact and continuous film formation. The adhesion strength, evaluated by the sellotape test, was found to be in the range of 80–88%, confirming good interfacial bonding between the polymer layer and the metallic substrate.

### 3.2. Electrochemical Analysis of the P3MPY–AOT/P3MTP/OL 37 Composite Coating

**Figure 3:**
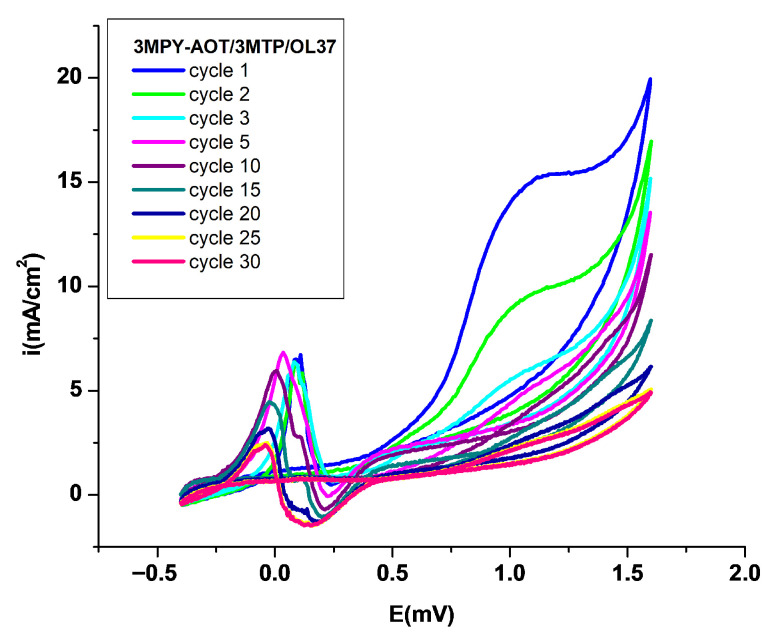
Cyclic voltammograms of the P3MPY–AOT/P3MTP/OL 37 composite films in 0.3 M C_2_H_2_O_4_ solution, recorded over the potential range –0.4 to +1.5 V vs. SCE at a scan rate of 20 mV/s.

Figure 3 manifests the cyclic voltammetry of P3MPY–AOT/P3MTP in 0.3 M H_2_C_2_O_4_ (−0.5 to +1.5 V vs. SCE, 20 mV/s), illustrating its electrochemical behavior. Cyclic voltammetry over an extended potential range was used to evaluate the full physical and electrochemical characteristics of the composite layer. Figure 3 shows that the coating’s electrochemical behavior depends on the cycles number and the electrodeposition parameters. The stability of a conductive polymer in both reduced and oxidized states is essential for many applications, with the inherent chemical stability of the polymer matrix being a key factor in its durability [8,22,26].

The lifetime of a conducting polymer is primarily determined by the intrinsic chemical stability of the polymer matrix. Cyclic voltammetry in oxalic acid (without monomer) shows that the P3MPY–AOT/P3MTP copolymeric films can be cycled many times between oxidized and reduced forms with minimal degradation. The current density decreases slightly over successive cycles before stabilizing, indicating excellent electrochemical stability.

### 3.3. FT-IR Spectroscopic Study

**Figure 4:**
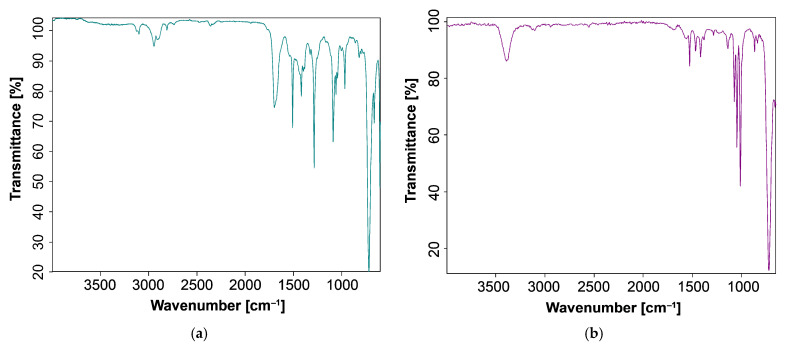

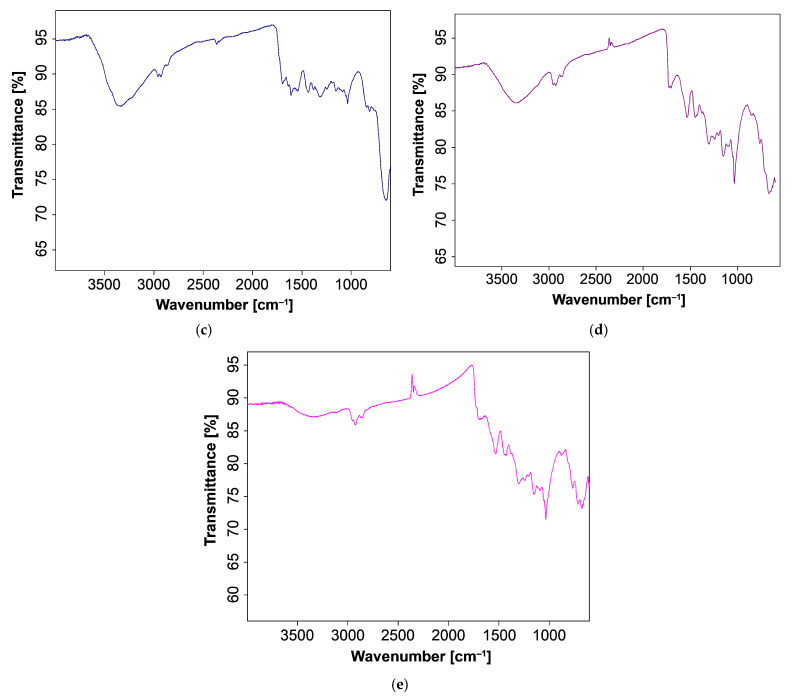
FT-IR spectra of (**a**) 3MPY, (**b**) 3MTP, and P3MPY–AOT/P3MTP/OL 37 obtained by electrochemical polymerization on OL 37 under potentiostatic and galvanostatic conditions: (**c**) at 1.0 V, (**d**) at 1.2 V, and (**e**) at 3 mA/cm^2^, using a 5:3 molar ratio of P3MPY–AOT and 3MTP.

The newly developed copolymer coatings were characterized by Fourier Transform Infrared (FT-IR) spectroscopy, as shown in Figure 4. The FT-IR analysis was employed to identify the characteristic functional groups and to elucidate the types of chemical bonding responsible for the formation of the novel polymer composites. The transmittance spectra of 3MPY, 3MTP, and the P3MPY–AOT/P3MTP/OL 37 coating exhibit the characteristic absorption peaks of the respective materials. The FT-IR spectra of the 3MPY and 3MTP monomers, presented in Figure 4a,b, respectively, exhibit characteristic absorption bands corresponding to their structural units. For 3MPY (Figure 4a), the distinct peaks observed at 1565 and 1424 cm^−1^ are assigned to the C=C stretching vibrations of the aromatic ring. Additional bands at 1397 and 2963 cm^−1^ correspond to the N–H stretching of the pyrrole ring and the C–H stretching of the 3-methylpyrrole groups, respectively. The peaks located at 1100, 1093, and 677 cm^−1^ are associated with in-plane and out-of-plane bending modes of C–H bonds within the polymer backbone [10,11,22,23,24,25,26]. In the case of 3MTP (Figure 4b), the absorption bands at 1680 and 1545 cm^−1^ are attributed to the C–C and C=C stretching vibrations of the quinoid structure in the thiophene ring. Vibrational bands characteristic of the sulfonate (SO_3_^−^) group is observed at 1244 and 1106 cm^−1^, corresponding to the asymmetric and symmetric stretching vibrations, respectively. Furthermore, the peaks appearing at 990, 833, and 661 cm^−1^ are assigned to C–S stretching vibrations of the thiophene ring [8,25,26,30].

The FT-IR spectra of the P3MPY–AOT/P3MTP/OL 37 coatings obtained by potentiostatic and galvanostatic deposition techniques are shown in Figure 4c–e. The absorption bands recorded at 3400 and 3200 cm^−1^ correspond to the N–H stretching vibrations of the polymer backbone, while the small peaks at 3500 and 3430 cm^−1^ are attributed to O–H stretching vibrations of the counterions. The bands appearing in the 3100–2909 cm^−1^ region are associated with the C–H stretching of the methyl groups in the 3-methylpyrrole units. The absorption band at 1261 cm^−1^ is assigned to the C–N stretching vibration of the pyrrole ring. Furthermore, characteristic aromatic C=C stretching vibrations are clearly evident at 1560 and 1420 cm^−1^, confirming the presence of conjugated aromatic structures within the P3MPY framework. The absorption bands located at 1588 and 1433 cm^−1^ correspond to the stretching vibrations of the quinoid rings (Figure 4c–e). The associated peaks at 1394 and 1324 cm^−1^ are attributed to the N–H stretching vibrations of the 3-methylpyrrole ring, while the bands appearing at 1690 and 1610 cm^−1^ are related to C=C stretching modes. In the P3MPY–AOT/P3MTP coatings, characteristic bands corresponding to asymmetric and symmetric C=C stretching vibrations of the quinoid structure in the thiophene ring are observed at 1561 and 1423 cm^−1^ (Figure 4c–e). Additionally, the absorption peaks at approximately 1078 and 776 cm^−1^ are assigned to the C–S–C stretching vibrations of the thiophene ring, whereas the band at 1521 cm^−1^ is correlated with the C=C stretching vibration [9,22,26,33]. The absorption bands observed at 1420 and 1321 cm^−1^ are attributed to the stretching vibrations of the CH_2_ and CH_3_ groups of the surfactant, while the peaks at 1078 and 660 cm^−1^ correspond to the S=O stretching vibrations of the AOT anionic surfactant. The appearance of C=O and C–H bonds is indicated by stretching vibrations at 1695 and 1246 cm^−1^, respectively, which are associated with the incorporation of the surfactant molecules into the polymer matrix (Figure 4c–e) [8,9,34]. In addition, the bands appearing in the 1042–767 cm^−1^ region correspond to in-plane and out-of-plane C–H vibrations of the aromatic rings, as well as to out-of-plane C–H bending modes in the P3MPY polymer doped with H_2_C_2_O_4_. A comparative analysis of Figure 4a,b and Figure 4c–e confirms that the P3MPY–AOT/P3MTP copolymer films were successfully deposited on the OL 37 steel surface. The observed FT-IR bands confirm the successful incorporation of AOT surfactant molecules into the P3MPY/P3MTP polymer matrix. The presence of CH_2_, CH_3_, and S=O stretching vibrations indicates that the surfactant is well-integrated within the composite, contributing to charge balance and enhanced ionic mobility. The retention of characteristic C=O, C–H, and aromatic C–H vibrations demonstrates that the monomeric structures of both MPY and MTP are preserved during co-deposition, ensuring that the conjugated polymer backbone remains intact. These features collectively suggest that AOT not only facilitates uniform film formation but also promotes strong interfacial adhesion between the polymer coating and the OL 37 steel substrate, leading to a chemically stable composite layer [9,26,34]. The characteristic absorption bands of both MPY and MTP monomeric units are clearly retained in the P3MPY–AOT/P3MTP films-coated samples spectra, indicating their effective incorporation into the polymeric structure and suggesting strong interfacial adherence of the coatings onto the metallic substrate.

### 3.4. Electrochemical Study of P3MPY–AOT/P3MTP/OL 37 Composite

#### 3.4.1. Potentiodynamic Polarization Analysis

**Figure 5:**
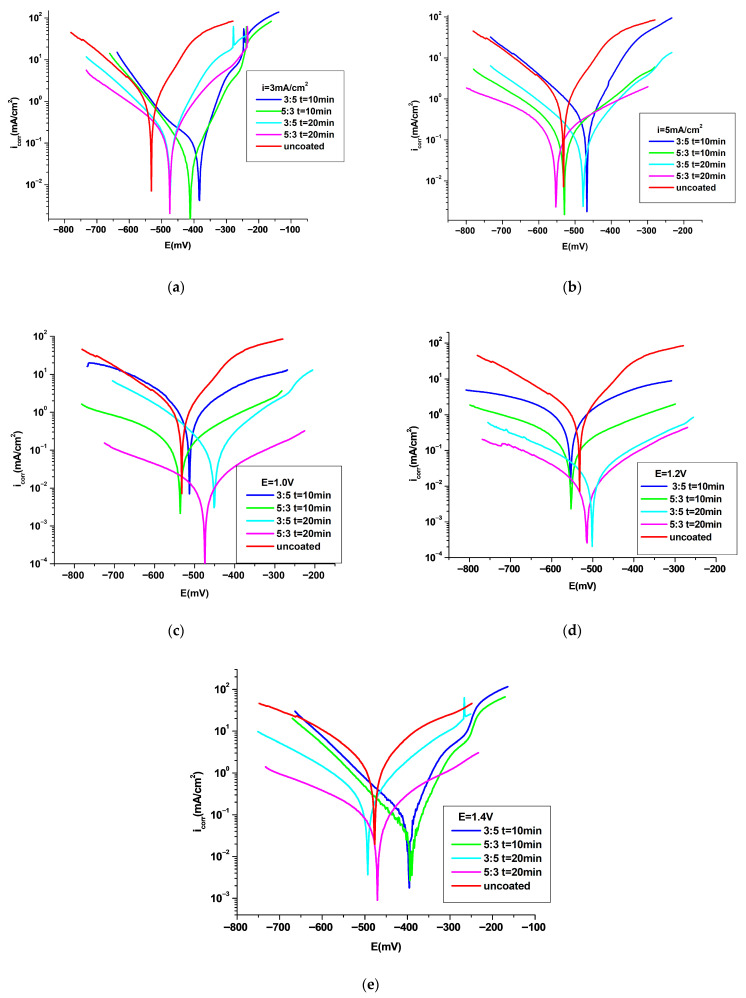
Polarization curves of P3MPY–AOT/P3MTP coated and uncoated over OL 37 substrate in 1 M HCl by galvanostatic at (**a**) 3 mA/cm^2^, (**b**) 5 mA/cm^2^ and at potentiostatic process at (**c**) 1.0 V, (**d**) 1.2 V and (**e**) 1.4 V potential applied and at some molar ratios for 10 and 20 min.

The corrosion resistance of the P3MPY–AOT/P3MTP/OL 37 composite in 1 M HCl was examined using potentiodynamic polarization and electrochemical impedance spectroscopy (EIS), providing insight into the protective behavior and interfacial stability of the polymer coating. Potentiodynamic polarization measurements were performed to investigate the corrosion kinetics and elucidate the reaction mechanisms occurring at the metal–electrolyte interface. The polarization curves, recorded in both the absence and presence of the polymeric composite in 1 M HCl solution, are presented in Figure 5. The Tafel polarization curves (Figure 5) revealed clear differences in the corrosion behavior between the bare OL 37 substrate and the P3MPY–AOT/P3MTP/OL 37 coated samples in 1 M HCl solution. The corresponding potentiodynamic polarization parameters are summarized in Table 1, Table 2 and Table 3. Figure 5 shows that the P3MPY–AOT/P3MTP/OL 37 composite coatings suppress both the anodic metal dissolution and the cathodic hydrogen evolution in aggressive media, indicating effective inhibition of the principal electrochemical reactions involved in corrosion. The significant decrease in corrosion current density, along with the increase in protection efficiency observed for the coated samples, demonstrates the enhanced corrosion resistance conferred by the polymer composite layer.

The bare OL 37 substrate exhibits the highest corrosion current density (i_corr_) and the most negative corrosion potential (E_corr_), indicating poor resistance to electrochemical degradation. Conversely, the application of the P3MPY-AOT/P3MTP coating (Figure 5) significantly reduced the icorr value and shifted Ecorr toward more positive potentials for most of the composite coatings (except a few), suggesting the formation of an effective barrier layer that limits charge transfer at the metal–electrolyte interface. For some coated samples, a slight shift of E_corr_ toward more negative values compared with the uncoated substrate may be attributed to changes in surface morphology and composition occurring during the electrodeposition process. Such modifications can locally disrupt the native passive film of the steel, leading to a decrease in E_corr_. This improvement in corrosion resistance can be attributed to the compact and adherent nature of the polymer film, which minimizes electrolyte penetration and provides enhanced corrosion inhibition through the synergistic interaction between 3-methylpyrrole–sodium dioctyl sulfosuccinate and 3-methylthiophene units within the copolymer matrix. This enhancement is likely due to the synergistic interaction between the conductive polymer network and the anionic surfactant AOT, which together form a compact, adherent, and hydrophobic protective layer. This layer effectively suppresses both anodic metal dissolution and cathodic oxygen reduction, thereby enhancing the overall corrosion resistance of the coated substrate [8,9,22,26,27].

The P3MPY–AOT/P3MTP coating exhibited remarkable corrosion protection, primarily due to the incorporation of AOT, a large anionic surfactant, into the P3MPY polymer matrix. The dopant not only enhanced the electrical conductivity of the polymer but also modulated ion selectivity, facilitating the formation of a uniform and compact protective layer. It is proposed that the long hydrocarbon chains of the surfactant competitively adsorb onto the carbon steel surface, thereby obstructing active corrosion sites and limiting the penetration of chloride ions (Cl^−^). Consequently, the corrosion rate of OL 37 steel coated with P3MPY–AOT/P3MTP was reduced by approximately nine-fold compared to bare OL 37, underscoring the coating’s effectiveness in mitigating the impact of aggressive species such as HCl. These findings highlight the potential of composite polymer–surfactant coatings in the development of advanced corrosion-resistant systems.

The corrosion behavior of bare OL 37 and P3MPY–AOT/P3MTP-coated OL 37 in 1 M HCl can be described by the following equations:

Anodic half-reaction:

dissolution of Metal (M = Fe)

M → M^n+^ + ne^−^

P3MPY_undoped_ − ne^−^ → P3MPY_doped_

P3MTP_undoped_ − ne^−^ → P3MTP_doped_

Cathodic half-reaction:

The oxygen reduction:

½ *O*_2_ + *H*_2_*O* + 2*e* → 2*HO*^−^

2H^+^ + 2e^−^ → H_2_

P3MPY_doped_ + ne^−^ → P3MPY_undoped_

P3MTP_doped_ + ne^−^ → P3MTP_undoped_

Chemical reactions:

M^2+^ + 2OH^−^ → M (OH)_2_ → M(OH)_3_ → M_2_O_3_

In acidic environments, the metal undergoes anodic dissolution, forming higher-valence species that dissolve in the medium, concomitantly reducing the hydrogen ions at the cathode, by electrons released from the metal.

**Figure 6:**
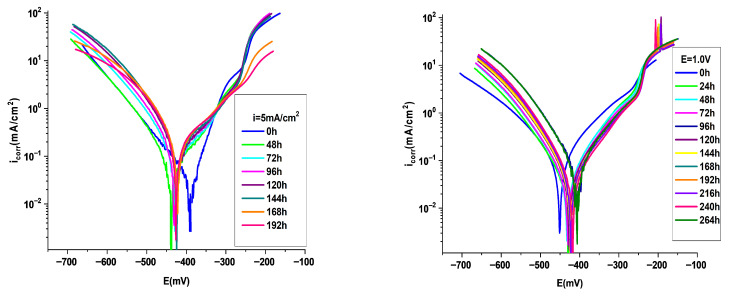
Polarization curves of P3MPY–AOT/P3MTP-coated OL 37 substrates in 1 M HCl, recorded under potentiostatic conditions at 1.0 V and galvanostatic conditions at 5 mA cm^−2^ for specific immersion times.

The protective performance of the P3MPY–AOT/P3MTP copolymer films after immersion periods is illustrated in Figure 6 and Table 3. Potentiodynamic polarization was employed to assess the effect of immersion time (0–196 h) on the corrosion inhibition of OL 37 steel in 1 M HCl. The results reveal a gradual decline in protective efficiency over time. Notably, after 100 h of immersion the corrosion rate was slightly increased. This reduction in protection is attributed to progressive alterations of the substrate surface and to minor imperfections within the coating, which facilitate the penetration of corrosive ions (chloride ions) to the OL 37/coating interface. The P3MPY–AOT/P3MTP/OL 37 polymer composite prepared via the potentiostatic method demonstrated markedly superior protection during long-term immersion, maintaining high efficiency for over 190 h, compared to the composite produced by the galvanostatic method. These findings highlight the importance of coating integrity in sustaining long-term corrosion resistance (in maintaining effective long-term corrosion resistance).

#### 3.4.2. Electrochemical Impedance Spectroscopy (EIS) Investigation

**Figure 7:**
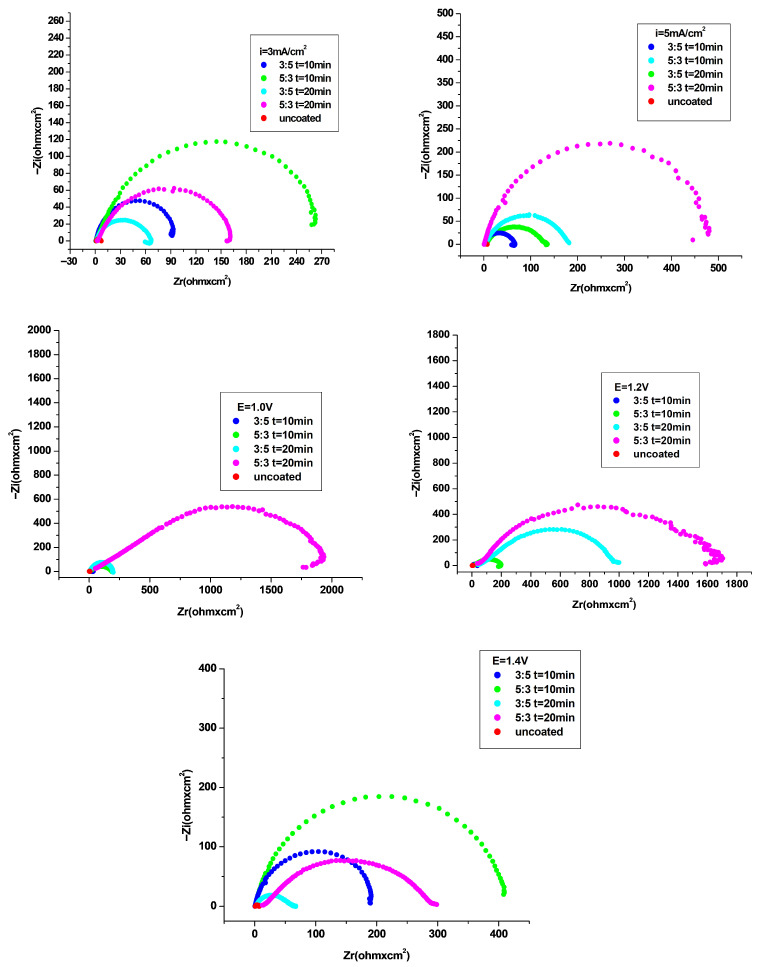
Nyquist plots of OL 37 and P3MPY–AOT/P3MTP coatings deposited by potentiostatic and galvanostatic methods at a specific molar ratio.

Electrochemical impedance spectroscopy (EIS) measurements were performed to elucidate the mechanisms governing the corrosion processes and to characterize the composite films formed on OL 37 substrates. This procedure enabled the evaluation of the corrosion protection offered by the P3MPY–AOT/P3MTP coatings under different conditions. Figure 7 illustrate the Nyquist impedance spectra recorded for the OL 37 substrate coated with P3MPY–AOT/P3MTP and for the uncoated OL 37 substrate immersed in HCl medium. As observed in Figure 7, all Nyquist plots display a single time constant, indicating a uniform electrochemical process. For the OL 37 electrode in HCl solution, the Nyquist plot displayed a small capacitive response, confirming that charge transfer dominates the corrosion process [8,9,22,26,29,30]. (The Nyquist plot of the uncoated OL 37 steel shows a small semicircular arc, which corresponds to a low charge transfer resistance and, consequently, poor corrosion resistance).

The presence of the P3MPY–AOT/P3MTP composite did not modify the semicircular profile, implying that the corrosion mechanism remains governed by charge transfer. Moreover, the progressive increase in the diameter of the semicircle with strengthening of the coating suggests enhanced corrosion resistance. Analysis of the Nyquist plots reveals that the impedance behavior of OL 37 was markedly influenced by the composite coatings, confirming that an effective protective layer was established using the P3MPY–AOT/P3MTP polymeric composite (see Figure 7 sand Table 4 and Table 5). The deviation of these capacitive loops from ideal semicircular shapes is commonly attributed to surface heterogeneity, which may result from surface roughness, the presence of impurities, crystal lattice defects, fractal characteristics, non-uniform distribution of active sites, and the formation of porous structures [22,26,27,28,30,33].

As illustrated in Figure 7, the capacitive loop diameters of the coatings obtained at applied potentials between 1.0, 1.2 and 1.4 V, current densities of 3 and 5 mA·cm^−2^, and molar ratios of 5:3 and 3:5 (for deposition times of 10 and 20 min) are significantly larger than those of the uncoated OL 37 substrate, indicating a pronounced protective effect of the composite coatings in corrosive media. From Figure 7, it is evident that the semicircle diameters of the P3MPY–AOT/P3MTP composite coatings obtained at applied potentials of 1.0, 1.2, and 1.4 V are larger than those obtained under galvanostatic conditions at current densities of 3 and 5 mA·cm^−2^ (for molar ratios of 5:3 and 3:5). Consequently, the protective performance of the composite coatings prepared potentiostatically is superior. Based on the experimental results presented in Figure 7 and Table 4 and Table 5, it can be inferred that the P3MPY–AOT/P3MTP coatings acted as an effective physical barrier, preventing the penetration of aggressive species into the composite layer, thereby reducing

**Figure 8:**
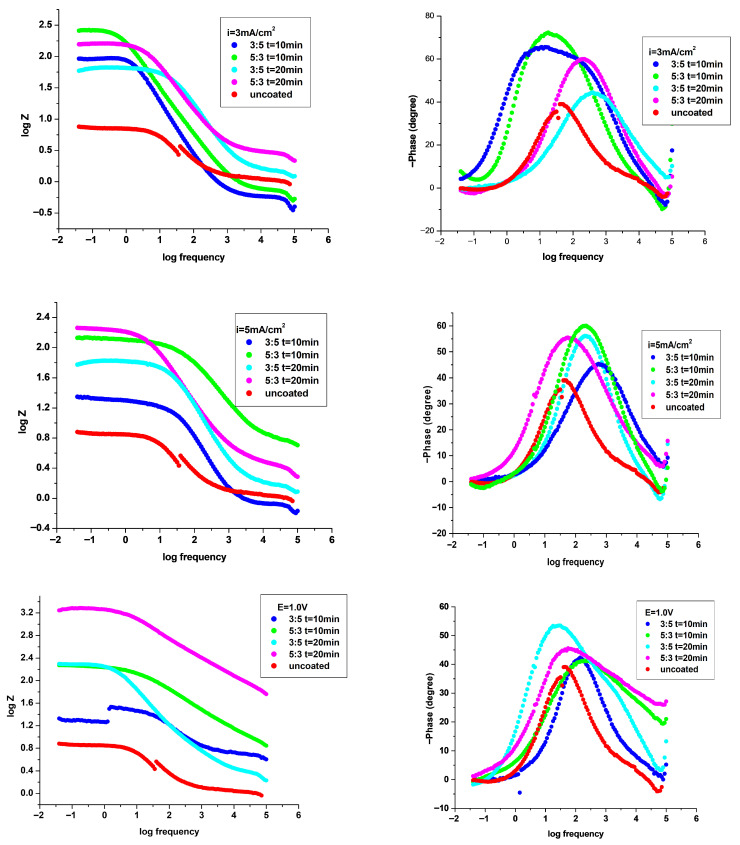

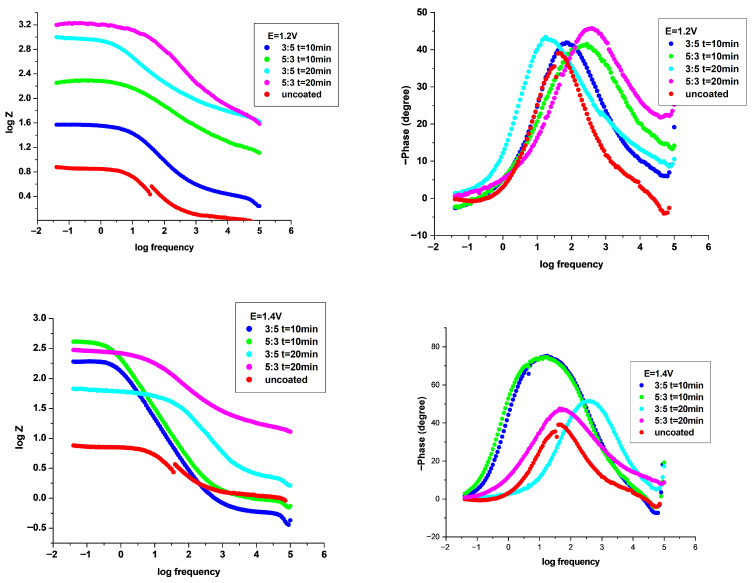
Bode plots of OL 37 and P3MPY–AOT/P3MTP coatings deposited by potentiostatic and galvanostatic methods at a specific molar ratio.

The Bode and phase angle plots presented in Figure 8 show that the phase angle values of the polymeric composite coatings are higher than those of the bare substrate. This indicates that the steel surface becomes smoother upon deposition of the composite coatings, confirming their effectiveness in reducing the surface roughness of OL 37 steel. Moreover, the phase angle values increase with the addition of consolidated composite material; however, they do not exceed 80°, suggesting a non-ideal capacitive behavior of the coatings. Furthermore, under these conditions, the covered OL 37 substrates show a good capacitive response, consistent with the Nyquist analysis and the findings from potentiodynamic polarization tests. In addition, the impedance magnitude at low frequencies in the presence of the coatings is significantly higher than that of the uncoated sample, indicating the formation of a continuous polymeric layer on the metal surface and demonstrating the excellent protective performance of the polymeric composite against corrosion of the OL 37 steel substrate. The increase in Z_mod_ indicates a significant protective effect, as the impedance magnitude rises with the improvement of the copolymer film. A higher impedance value is associated with enhanced corrosion resistance.

To gain deeper insight into the investigated system and the electrochemical processes at the substrate/electrolyte interface, the EIS and Bode results were analyzed using the equivalent electrical circuits presented below.

**Figure 9:**
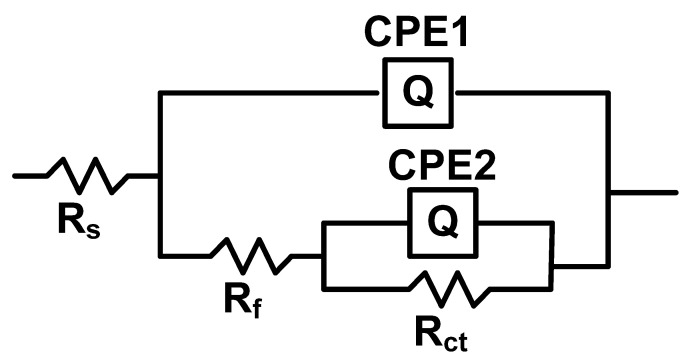
Equivalent circuit.

The electrochemical impedance spectroscopy (EIS) data recorded for the P3MPY–AOT/P3MTP-coated OL 37 steel in 1 M HCl were analyzed by fitting to the equivalent circuit presented in Figure 9. In this circuit, Rs denotes the solution resistance, Rf the resistance of the coating film, and Cf its corresponding capacitance. The charge transfer resistance (Rct) is arranged in parallel with a constant phase element (CPE), which represents the double-layer capacitance (Cdl). All electrochemical parameters, together with the calculated protection efficiency and chi-square (χ^2^) values, are presented in Table 4 and Table 5. The impedance of the constant phase element (CPE), which accounts for the non-ideal capacitive behavior arising from surface heterogeneity, is expressed as: Z_CPE_ = Y_0_^−1^ (jω)^−n^.

In this expression, ω denotes the angular frequency, j is the imaginary unit (j^2^ = −1), Y_0_ represents the CPE magnitude, and n is the phase shift exponent. The value of n provides insight into the surface homogeneity: the closer n is to 1, the smoother and more uniform the surface. The CPE behaves as a resistor when n = 0 (Y_0_ = R), as a capacitor when n = 1 (Y_0_ = C), as an inductor when n = −1 (Y_0_ = L), and as a Warburg impedance when n = 0.5 (Y_0_ = W). Electrochemical impedance spectroscopy (EIS) results reveal that the application of P3MPY–AOT/P3MTP composite coatings increases the charge transfer resistance (Rct) while decreasing the double-layer capacitance (Cdl). The observed increase in Rct with the composite coatings corresponds to a marked improvement in protective performance, indicating that the coating provides substantial corrosion inhibition for OL 37 steel. The decrease in Cdl is likely due to a reduction in the local dielectric constant and/or an increase in the thickness of the electrical double layer, arising from the adsorption of the polymeric composite at the OL 37/HCl interface. The P3MPY–AOT/P3MTP coating applied to the OL 37 steel substrate by electropolymerization forms an effective protective layer. Analysis of the Nyquist and Bode diagrams (Figure 7 and Figure 8, Table 4 and Table 5) demonstrates that the composite coating suppresses the corrosion process, primarily by acting as a diffusion barrier that impedes charge transfer at the interface. These observations are in good agreement with the Nyquist plots, further confirming the improved. These electrochemical impedance results are consistent with the polarization measurements, supporting the conclusion that the P3MPY–AOT/P3MTP composite coatings effectively hinder charge transfer and enhance the corrosion resistance of OL 37 steel.

**Figure 10:**
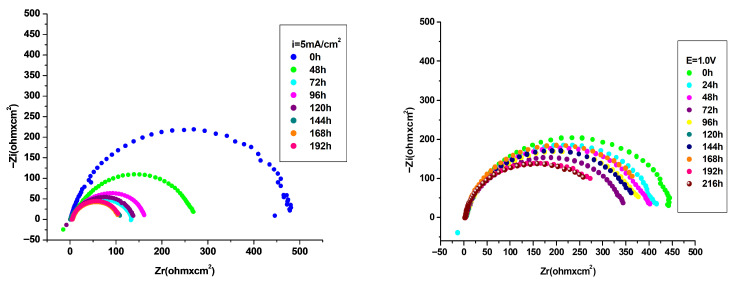
Nyquist plots of P3MPY–AOT/P3MTP/OL 37-coated specimens prepared by electrochemical deposition, recorded at different immersion times.

**Figure 11:**
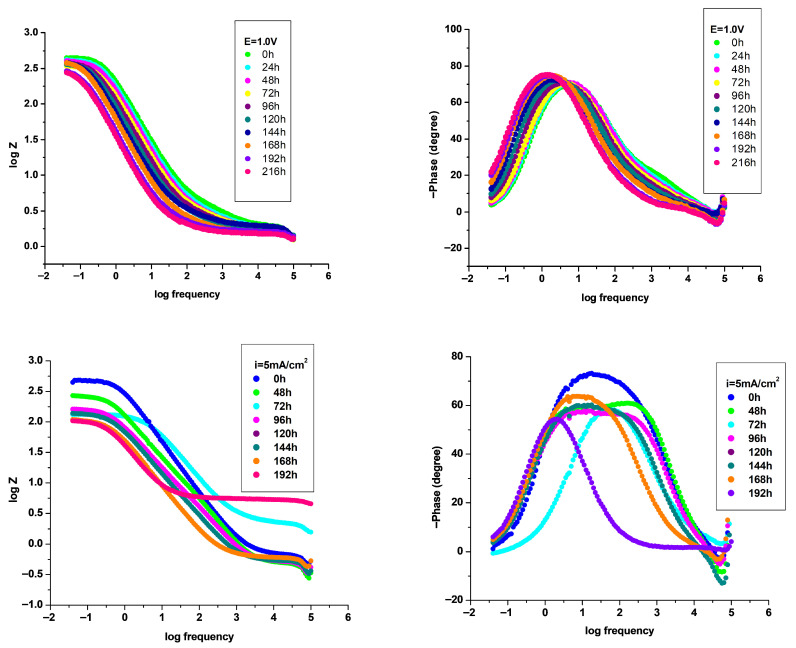
Bode plots of P3MPY–AOT/P3MTP/OL 37-coated specimens prepared by electrochemical deposition, recorded at different immersion times.

As shown in Figure 10, the diameter of the capacitive loops for the P3MPY–AOT/P3MTP-coated samples decrease slightly with increasing immersion time. A minor decline in protective efficiency between 96 and 192 h indicates the diffusion of chloride ions (Cl^−^) into the coating. Nevertheless, the EIS results confirm that the P3MPY–AOT/P3MTP coating maintains effective resistance to the ingress of aggressive species over prolonged immersion periods. The variation of the impedance magnitude and phase angle with immersion time, as observed in the Bode plots (Figure 11), provides valuable insight into the stability of the composite protective films. During prolonged immersion, films obtained by the potentiostatic deposition method exhibit only a slight decrease in impedance at low frequencies and maintain a relatively stable phase angle, indicating the preservation of their capacitive behavior and structural integrity. Comparatively, coatings prepared by the galvanostatic method show a slightly lower impedance and a tiny shift in the phase angle, suggesting increased surface heterogeneity and degradation of the protective layer. These observations confirm that potentiostatically deposited films possess a more compact and adherent structure, which enhances their corrosion resistance and long-term stability in the electrolyte environment.

### 3.5. SEM-EDX Study

**Figure 12:**
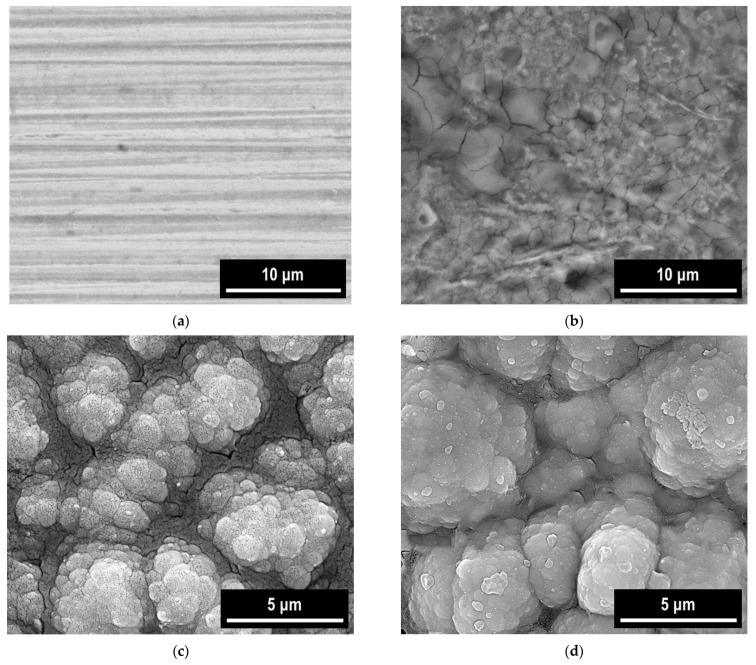

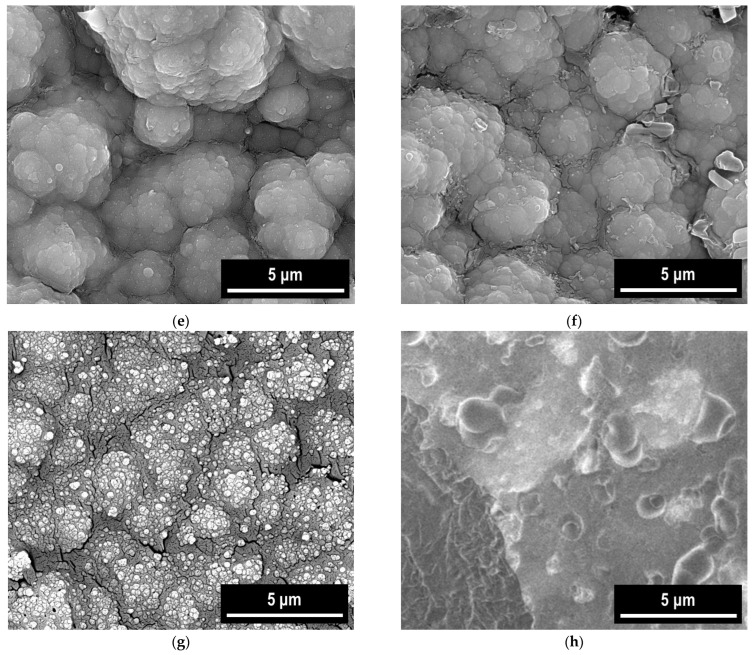
SEM micrographs of OL 37 substrates coated with P3MPY–SDS/PEDOT: (**a**) uncoated substrate; (**b**) substrate exposed to 1 M HCl; (**c**) coating deposited under galvanostatic conditions at 5 mA/cm^2^; (**d**–**f**) coatings obtained under potentiostatic control at applied potentials of 1.0, 1.2, and 1.4 V with 5:3 and 3:5 molar ratios; (**g**,**h**) surface morphology after 72 h and 196 h immersion in 1 M HCl.

**Figure 13:**
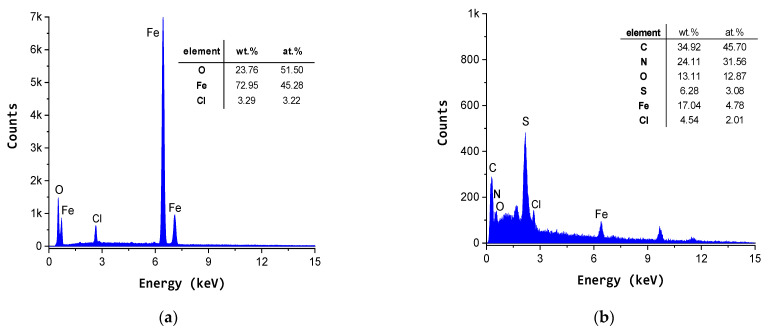

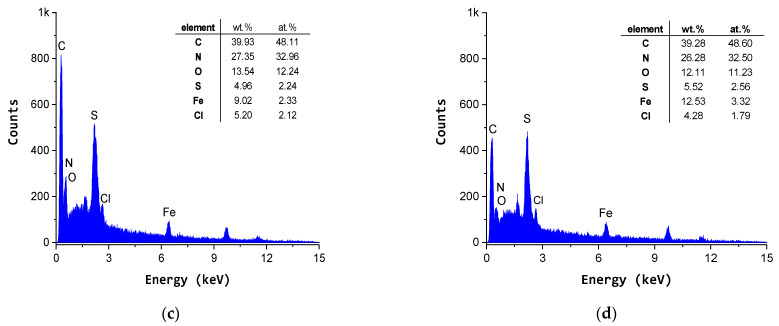
EDS spectra corresponding to uncoated and coated OL 37 samples. (**a**) uncoated substrate exposed to 1 M HCl; (**b**) coating deposited under galvanostatic conditions at 5 mA/cm^2^; (**c**) coatings obtained under potentiostatic control at applied potentials of 1.0V; (**d**) at applied potentials of 1.2V.

The surface morphology of P3MPY–AOT/P3MTP coatings on OL 37 steel was investigated by electron microscopy. Representative SEM images of the coatings electrodeposited under different conditions are shown in Figure 12. Both potentiostatic and galvanostatic deposition methods produced homogeneous and dense black composite layers on the OL 37 substrates (Figure 12c–f). The coatings display a granular, cauliflower-like morphology with a uniform distribution of particles and minor size variations, in agreement with previously reported studies [8,26,33,36,37,38]. The SEM micrographs of P3MPY–AOT/P3MTP coatings demonstrate the formation of a dense and adherent film onto the carbon steel surface, free from visible cracks, reflecting the high quality of the deposited layer. Incorporation of the anionic surfactant AOT as a dopant into the conducting polymer plays a determinant role in the electrodeposition process and in enhancing the properties of the resulting composite coating. The superior quality of the coating and its enhanced adsorption properties contribute to the outstanding protective performance of the composite layer [8,26,33]. EDS (energy-dispersive X-ray spectroscopy) analysis of the P3MPY–AOT/P3MTP-coated and uncoated carbon steel substrate was carried out, with the resulting spectra shown in Figure 13a–d. The EDX analysis of the OL 37 carbon steel sample after immersion in HCl solution shows prominent peaks for chloride (Cl) and oxygen (O) (Figure 13a), reflecting the formation of corrosion products on the substrate surface. The presence of the protective film on OL 37 is evidenced by the C, N, O, and S peaks in the EDS spectra (Figure 13b–d) [8,9,22,23,24,25,26,36,37,38]. These findings are in agreement with FTIR data, confirming the incorporation of the surfactant and oxalate ions into the polymer matrix of the coating.

The application of SEM–EDS analyses allowed for a comprehensive evaluation of both morphological and compositional modifications on the specimens’ surfaces, providing essential insights into the impact of environmental exposure and the material’s performance under simulated conditions. After immersion in acidic medium for up to 200 h, the composite exhibited discernible morphological changes, as corroborated by electrochemical measurements. SEM micrographs (Figure 12g,h) indicate penetration of chloride ions (Cl^−^) into the P3MPY–AOT/P3MTP layer. Furthermore, elongated fragments, likely residual polymerization by-products or surface deposits, were observed, which could affect the coating’s homogeneity and electrochemical behavior. These morphological alterations are primarily associated with immersion-induced slight degradation and deposition on the coating surface.

## 4. Conclusions

SEM observations, electrochemical analyses, and adhesion tests confirm the successful electrodeposition of uniform, compact, and strongly adherent P3MPY–AOT/P3MTP composite coatings on OL 37 steel from oxalic acid solutions. Overall, the results demonstrate that electrodeposition offers a reliable, efficient, and cost-effective approach for producing protective polymeric films, significantly enhancing the corrosion resistance of steel substrates.

Electrochemical investigations revealed that the P3MPY–AOT/P3MTP composite coating provides efficient corrosion protection for OL 37 steel in hydrochloric acid solution. Optimization of the coating composition and electrodeposition parameters further improved its protective efficiency, as confirmed by potentiodynamic polarization and electrochemical impedance spectroscopy performed in 1 M HCl.

The P3MPY–AOT/P3MTP coating on OL 37 steel reduces the corrosion rate by nearly an order of magnitude compared to the uncoated substrate, achieving a protection efficiency exceeding 90%. The observed corrosion-resistance trend—P3MPY–AOT/P3MTP deposited at 1.2 V > 1.0 V > 1.4 V > 3 mA·cm^−2^ > 5 mA·cm^−2^—highlights the significant influence of deposition parameters on coating performance and confirms the composite’s remarkable ability to mitigate corrosion processes. Overall, these results validate the superior protective behavior and long-term stability of the composite coating in acidic environments.

FT-IR analysis confirms the successful formation of the P3MPY–AOT/P3MTP composite layer on the OL 37 substrate. The resulting coating exhibits a compact, uniform morphology with low porosity and strong barrier characteristics. The P3MPY–AOT/P3MTP films deposited potentiostatically at 1.2 V (5:3, t = 20 min) demonstrate significantly superior protective performance compared to those obtained at 1.4 V, whereas galvanostatic deposition at 3 mA/cm^2^ (5:3, t = 10 and 20 min) provides higher protection efficiency than deposition at 5 mA/cm^2^. These findings, supported by the experimental data, highlight the increased surface coverage and adsorption effectiveness, which enhance the corrosion resistance of the coating

Composite coatings electrodeposited by the potentiostatic method exhibited superior corrosion protection compared to those obtained by the galvanostatic method. Furthermore, coatings produced using a 5:3 molar ratio demonstrated enhanced protective performance relative to those deposited with a 3:5 molar ratio.

These electrochemical results are consistent with the surface morphology observations and the electrochemical impedance spectroscopy data, both of which further confirm the enhanced protective performance and improved film integrity of the P3MPY–AOT/P3MTP coating on the OL 37 substrate.

The copolymer coatings prepared by the specified parameters effectively inhibit the corrosion of the carbon steel substrate. Therefore, the electrochemically synthesized P3MPY–AOT/P3MTP composite exhibits significant potential for practical application in the protection of a wide range of metallic materials.

The excellent anticorrosion protection of the newly electro-synthesized conducting polymers polypyrrole, polythiophene and their derivatives based composite coatings makes them promising candidates for further enhancement using state-of-the-art strategies. Incorporating functionalized or non-functionalized carbon nanotubes, graphene, surfactants, or metal oxides could improve layer compactness, electrical conductivity, and mechanical strength, further boosting their protective performance.

## Figures and Tables

**Table 1:** Kinetic corrosion parameters of coated and uncoated of OL 37 electrode in 1 M HCl solutions at 25 °C.

Coating System	−E_corr_ (mV)	i_corr_ (mA/cm^2^)	R_p_ (Ωcm)	R_mpy_	P_mm/year_	Kg (g/m^2^h)	b_a_ (mV/ Decade)	−b_c_ (mV/ Decade)	E (%)
OL 37 + 1 M HCl	529	0.873	26	409	10.39	9.29	88	115	-
P3MPY–AOT/P3MTP 3 mA/cm^2^ 3:5 molar ratio, t = 10 min	401	0.057	179	26.6	0.675	0.60	49	102	93
P3MPY–AOT/P3MTP 3 mA/cm^2^ 5:3 molar ratio, t = 10 min	415	0.030	350	14	0.355	0.32	60	71	97
P3MPY–AOT/P3MTP 3 mA/cm^2^ 3:5 molar ratio, t = 20 min	480	0.115	116	53.66	1.36	1.22	61	99	87
P3MPY–AOT/P3MTP 3 mA/cm^2^ 5:3 molar ratio, t = 20 min	485	0.098	139	45.98	1.16	1.06	73	106	90
P3MPY–AOT/P3MTP 5 mA/cm^2^ 3:5 molar ratio, t = 10 min	470	0.19	64	89.15	2.26	2.02	100	94	78
P3MPY–AOT/P3MTP 5 mA/cm^2^ 5:3 molar ratio, t = 10 min	526	0.081	211	38	0.964	0.86	105	100	91
P3MPY–AOT/P3MTP 5 mA/cm^2^ 3:5 molar ratio, t = 20 min	480	0.091	182	42.7	1.083	0.97	100	94	90
P3MPY–AOT/P3MTP 5 mA/cm^2^ 5:3 molar ratio, t = 20 min	532	0.064	339	30.03	0.762	0.68	75	90	93

**Table 2:** Kinetic corrosion parameters of coated and uncoated of OL 37 electrode in 1 M HCl solutions at 25 °C.

Coating System	−E_corr_ (mV)	i_corr_ (mA/cm^2^)	R_p_ (Ωcm)	R_mpy_	P_mm/year_	Kg (g/m^2^h)	b_a_ (mV/ Decade)	−b_c_ (mV/ Decade)	E (%)
OL 37 + 1 M HCl	529	0.873	26	409	10.39	9.29	88	115	-
P3MPY–AOT/P3MTP 1.0 V 3:5 molar ratio, t = 10 min	513	0.34	61	159.3	4.06	3.62	92	82	61
P3MPY–AOT/P3MTP 1.0 V 5:3 molar ratio, t = 10 min	521	0.075	225	35.19	0.893	0.799	94	106	91
P3MPY–AOT/P3MTP 1.0 V 3:5 molar ratio, t = 20 min	450	0.063	220	29.26	0.74	0.60	78	86	93
P3MPY–AOT/P3MTP 1.0 V 5:3 molar ratio, t = 20 min	490	0.006	2580	3.28	0.083	0.07	85	112	99
P3MPY–AOT/P3MTP 1.2 V 3:5 molar ratio, t = 10 min	515	0.25	65	117.3	2.977	2.66	90	102	71
P3MPY–AOT/P3MTP 1.2 V 5:3 molar ratio, t = 10 min	523	0.069	216	32.37	0.822	0.73	102	93	92
P3MPY–AOT/P3MTP 1.2 V 3:5 molar ratio, t = 20 min	516	0.014	1120	6.57	0.166	0.15	93	101	98
P3MPY–AOT/P3MTP 1.2 V 5:3 molar ratio, t = 20 min	512	0.006	2690	2.81	0.071	0.06	84	98	99
P3MPY–AOT/P3MTP 1.4 V 3:5 molar ratio, t = 10 min	400	0.075	163	35.19	0.893	0.79	50	99	91
P3MPY–AOT/P3MTP 1.4 V 5:3 molar ratio, t = 10 min	410	0.031	360	14.54	0.369	0.33	49	91	97
P3MPY–AOT/P3MTP 1.4 V 3:5 molar ratio, t = 20 min	499	0.151	98	70.85	1.798	1.61	75	93	83
P3MPY–AOT/P3MTP 1.4 V 5:3 molar ratio, t = 20 min	520	0.076	198	35.66	0.905	0.81	97	98	90

**Table 3:** Kinetic corrosion parameters of coated and uncoated of OL 37 electrode in 1 M HCl solutions at 25 °C at different immersion times.

Coating System	−E_corr_ (mV)	i_corr_ (mA/cm^2^)	R_p_ (Ωcm)	R_mpy_	P_mm/year_	Kg (g/m^2^h)	b_a_ (mV/ Decade)	−b_c_ (mV/ Decade)	E (%)
OL 37 + 1 M HCl	529	0.873	26	409	10.39	9.29	88	115	-
P3MPY–AOT/P3MTP 1.0 V 3:5 molar ratio, t = 20 min 0 h	432	0.026	495	12.13	0.333	0.275	66	72	97
P3MPY–AOT/P3MTP 1.0 V 3:5 molar ratio, t = 20 min 24 h	434	0.034	387	15.86	0.402	0.36	69	72	96
P3MPY–AOT/P3MTP 1.0 V 3:5 molar ratio, t = 20 min 48 h	433	0.033	404	15.4	0.391	0.35	71	71	96
P3MPY–AOT/P3MTP 1.0 V 3:5 molar ratio, t = 20 min 72 h	410	0.064	217	29.86	0.758	0.6378	81	74	93
P3MPY–AOT/P3MTP 1.0 V 3:5 molar ratio, t = 20 min 96 h	430	0.036	361	16.8	0.426	0.381	74	69	96
P3MPY–AOT/P3MTP 1.0 V 3:5 molar ratio, t = 20 min 120 h	427	0.037	373	17.26	0.438	0.392	75	70	95
P3MPY–AOT/P3MTP 1.0 V 3:5 molar ratio, t = 20 min 144 h	425	0.038	363	17.73	0.451	0.403	78	68	95
P3MPY–AOT/P3MTP 1.0 V 3:5 molar ratio, t = 20 min 168 h	426	0.0385	353	17.96	0.456	0.41	80	67	95
P3MPY–AOT/P3MTP 1.0 V 3:5 molar ratio, t = 20 min 192 h	421	0.0375	375	16.70	0.426	0.380	80	66	95
P3MPY–AOT/P3MTP 1.0 V 3:5 molar ratio, t = 20 min 216 h	432	0.026	495	12.13	0.333	0.275	66	72	97
P3MPY–AOT/P3MTP 1.0 V 3:5 molar ratio, t = 20 min 240 h	410	0.0576	234	26.88	0.682	0.61	77	70	93
P3MPY–AOT/P3MTP 5 mA/cm^2^ 5:3 molar ratio, t = 20 min 0 h	480	0.064	215	30.03	0.762	0.68	75	90	93
P3MPY–AOT/P3MTP 5 mA/cm^2^ 5:3 molar ratio, t = 20 min 48 h	440	0.066	287	26.77	0.66	0.61	106	71	93
P3MPY–AOT/P3MTP 5 mA/cm^2^ 5:3 molar ratio, t = 20 min 72 h	431	0.087	177	40.6	1.03	0.922	114	70	90
P3MPY–AOT/P3MTP 5 mA/cm^2^ 5:3 molar ratio, t = 20 min 96 h	432	0.111	148	51.8	1.314	1.17	119	70	87
P3MPY–AOT/P3MTP 5 mA/cm^2^ 5:3 molar ratio, t = 20 min 120 h	427	0.130	129	60.66	1.54	1.38	118	68	87
P3MPY–AOT/P3MTP 5 mA/cm^2^ 5:3 molar ratio, t = 20 min 144 h	427	0.14	108	65.33	1.658	1.484	120	66	84
P3MPY–AOT/P3MTP 5 mA/cm^2^ 5:3 molar ratio, t = 20 min 168 h	425	0.16	100	74.66	1.89	1.69	114	71	82
P3MPY–AOT/P3MTP 5 mA/cm^2^ 5:3 molar ratio, t = 20 min 196 h	428	0.17	100	79.33	2.01	1.80	121	74	81

**Table 4:** Electrochemical impedance spectroscopy (EIS) parameters for coated and uncoated OL 37 in 1 M HCl solution at 25 °C.

Coating System	Rs ohm·cm^2^	Q-Yo S·s^−n^·cm^−2^	Q-n	Rf ohm·cm^2^	Q-Yo S·s^−n^·cm^−2^	Q-n	Rct ohm·cm^2^	χ
OL 37 + 1 M HCl	0.77	0.0026	0.68	1.74	0.00518	0.72	9.34	4.645 × 10^−3^
P3MPY–AOT/P3MTP 1.0 V 3:5 molar ratio, t = 10 min	5.70	0.000133	0.70	29	0.000236	0.72	128	9.645 × 10^−5^
P3MPY–AOT/P3MTP 1.0 V 5:3molar ratio, t = 10 min	8.4	0.000101	072	36	0.000189	0.76	162	4.33 × 10^−4^
P3MPY–AOT/P3MTP 1.0 V 3:5 molar ratio, t = 20 min	2.29	0.000206	0.78	14	0.000253	0.82	191	1.089 × 10^−3^
P3MPY–AOT/P3MTP 1.0 V 5:3 molar ratio, t = 20 min	41	0.000022	0.71	460	0.000019	0.78	1608	4.269 × 10^−4^
P3MPY–AOT/P3MTP 1.2 V 3:5 molar ratio, t = 10 min	2.28	0.000266	0.76	12	0.000674	0.77	38	4.024 × 10^−4^
P3MPY–AOT/P3MTP 1.2 V 5:3molar ratio, t = 10 min	12	0.000093	0.73	38	0.000177	0.76	166	7.882 × 10^−4^
P3MPY–AOT/P3MTP 1.2 V 3:5 molar ratio, t = 20 min	45	0.000045	0.75	118	0.000058	0.78	841	7.494 × 10^−4^
P3MPY–AOT/P3MTP 1.2 V 5:3 molar ratio, t = 20 min	32	0.000021	0.73	201	0.000011	0.91	1478	3.280 × 10^−4^
P3MPY–AOT/P3MTP 1.4 V 3:5molar ratio, t = 10 min	0.63	0.000547	0.94	18	0.000448	0.92	198	8.414 × 10^−4^
P3MPY–AOT/P3MTP 1.4 V 5:3 molar ratio, t = 10 min	0.95	0.000338	0.93	22	0.000396	0.90	420	7.318 × 10^−4^
P3MPY–AOT/P3MTP 1.4 V 3:5 molar ratio, t = 20 min	2.17	0.000183	0.81	16	0.000814	0.78	61	1.313 × 10^−3^
P3MPY–AOT/P3MTP 1.4 V 5:3 molar ratio, t = 20 min	12	0.000252	0.71	39	0.000732	0.77	265	1.379 × 10^−4^

**Table 5:** Electrochemical impedance spectroscopy (EIS) parameters for coated and uncoated OL 37 in 1 M HCl solution at 25 °C.

Coating System	Rs ohm·cm^2^	Q-Yo S·s^−n^·cm^−2^	Q-n	Rf ohm·cm^2^	Q-Yo S·s^−n^·cm^−2^	Q-n	Rct ohm·cm^2^	χ
OL 37 + 1 M HCl	0.77	0.0026	0.68	1.74	0.00518	0.72	9.34	4.645 × 10^−3^
P3MPY–AOT/P3MTP 3 mA/cm^2^ 3:5 molar ratio, t = 10 min	0.59	0.000589	0.95	12	0.000398	0.94	94	1.752 × 10^−3^
P3MPY–AOT/P3MTP 3 mA/cm^2^ 5:3molar ratio, t = 10 min	0.73	0.000697	0.88	28	0.000236	0.92	260	1.490 × 10^−3^
P3MPY–AOT/P3MTP 3 mA/cm^2^ 3:5 molar ratio, t = 20 min	1.513	0.000036	0.93	18	0.000214	0.78	66	1.032 × 10^−3^
P3MPY–AOT/P3MTP 3 mA/cm^2^ 5:3 molar ratio, t = 20 min	2.886	0.000234	0.85	21	0.000142	0.83	153	6.020 × 10^−4^
P3MPY–AOT/P3MTP 5 mA/cm^2^ 3:5 molar ratio, t = 10 min	0.84	0.000433	0.89	12	0.000486	0.76	68	1.657 × 10^−3^
P3MPY–AOT/P3MTP 5 mA/cm^2^ 5:3 molar ratio, t = 10 min	2.5	0.000112	0.82	14	0.000116	0.81	178	2.406 × 10^−4^
P3MPY–AOT/P3MTP 5 mA/cm^2^ 3:5 molar ratio, t = 20 min	5.5	0.000103	0.73	23	0.000331	0.69	121	1.682 × 10^−4^
P3MPY–AOT/P3MTP 5 mA/cm^2^ 5:3 molar ratio, t = 20 min	0.681	0.000379	0.89	16	0.000125	0.89	487	1.228 × 10^−3^

## Data Availability

The original contributions presented in this study are included in the article. Further inquiries can be directed to the corresponding author.

## References

[1] Bouabdallaoui M., Aouzal Z., El Guerraf A., Ben Jadi S., Bazzaoui M., Wang R., Bazzaoui E.A. (2020). Influence of Polythiophene Overoxidation on Its Physicochemical Properties and Corrosion Protection Performances. Mater. Today Proc..

[2] Jiang L., Syed J.A., Lu H., Meng X. (2019). In-Situ Electrodeposition of Conductive Polypyrrole-Graphene Oxide Composite Coating for Corrosion Protection of 304SS Bipolar Plates. J. Alloys Compd..

[3] Li J., Liu J.-C., Gao C.-J. (2010). On the mechanism of conductivity enhancement in PEDOT/PSS film doped with multi-walled carbon nanotubes. J. Polym. Res..

[4] Branzoi F., Băran A., Mihai M.A., Zaki M.Y. (2024). The Inhibition Action of Some Brij-Type Nonionic Surfactants on the Corrosion of OLC 45 in Various Aggressive Environments. Materials.

[5] Luo Y., Song Y., Ma J., Yang J., Chen D., Gong H., Li G.L. (2025). Intrinsic Self-Healing Polymer Coatings with Autonomous Corrosion Protection Based on Synergistic Healing Mechanisms. Appl. Surf. Sci..

[6] (2022). Nimet Ceren Güven, Hatice Ozkazanc, Corrosion protection behavior of poly (Nmethylpyrrole)/boron nitride composite film on aluminum-1050. Prog. Org. Coat..

[7] Sarıarslan H., Karaca E., Şahin M., Pekmez N.Ö. (2020). Electrochemical Synthesis and Corrosion Protection of Poly(3-Aminophenylboronic Acid-Co-Pyrrole) on Mild Steel. RSC Adv..

[8] Branzoi F., Mihai A.M., Zaki M.Y. (2024). Anticorrosion Protection of New Composite Coating for Cobalt-Based Alloy in Hydrochloric Acid Solution Obtained by Electrodeposition Methods. Coatings.

[9] Pekmez N.Ö., Cınkıllı K., Zeybek B. (2014). The electrochemical copolymerization of pyrrole and bithiophene on stainless steel in the presence of SDS in aqueous medium and its anticorrosive performance. Prog. Org. Coat..

[10] Gopi D., Saraswathy R., Kavitha L., Kim D.-K. (2014). Electrochemical synthesis of poly(indole-co-thiophene) on low-nickel stainless steel and its anticorrosive performance in 0.5 mol L^−1^ H_2_SO_4_. Polym. Int..

[11] Kanwal S., Akhter Z., Ali N.Z., Hussain R., Qamar S. (2022). Corrosion Protection of Aluminum Alloy (AA2219-T6) Using Sulfonic Acid-Doped Conducting Polymer Coatings. New J. Chem..

[12] Ramírez A.M.R., Mieres F., Pineda F., Grez P., Heyser C. (2022). Electrosynthesis of polyindole-carboxylic acids on stainless steel and their corrosion protection at different temperatures in acidic solution. Prog. Org. Coat..

[13] Kausar A., Ahmad I., Eisa M.H., Maaza M. (2023). Avant-Garde Polymer/Graphene Nanocomposites for Corrosion Protection: Design, Features, and Performance—A Review. Corros. Mater. Degrad..

[14] Saikia M., Dutta T., Jadhav N., Kalita D.J. (2025). Insights into the Development of Corrosion Protection Coatings—A Review. Polymers.

[15] Sun Y., Hu C., Cui J., Shen S., Qiu H., Li J. (2022). Electrodeposition of polypyrrole coatings doped by benzenesulfonic acid-modified graphene oxide on metallic bipolar plates. Prog. Org. Coat..

[16] Cao G., Teng Z., Wu A., Zhang C., Wang Z., Wang L., Han E. (2025). Enhanced Corrosion Protection of Waterborne Polyurethane Coating Using Highly Conductive and Dispersed Polythiophene Nanoparticles. Polym. Degrad. Stab..

[17] Yağan A., Pekmez N., Yıldız A. (2007). Poly(N-methylaniline) coatings on stainless steel by electropolymerization. Corros. Sci..

[18] Yılmaz S.M., Atun G. (2022). Corrosion protection efficiency of the electrochemically synthesized polypyrrole-azo dye composite coating on stainless steel. Prog. Org. Coat..

[19] Qiao M., Ji G., Lu Y., Zhang B. (2023). Sustainable Corrosion-Resistant Superhydrophobic Composite Coating with Strengthened Robustness. J. Ind. Eng. Chem..

[20] Song L., Gao Z., Sun Q., Chu G., Shi H., Xu N., Li Z., Hao N., Zhang X., Ma F. (2023). Corrosion protection performance of a coating with 2-aminino-5-mercato-1,3,4-thiadizole-loaded hollow mesoporous silica on copper. Prog. Org. Coat..

[21] Luo Y., Li X., Luo Z., Chen L., Han G. (2023). Enhanced adhesive and anti-corrosive performances of polymer composite coating for rusted metallic substrates by capillary filling. Prog. Org. Coat..

[22] Raheem H.M., Salman T.A., Jabor A.A. (2025). Anti-Corrosion Behavior of Copolymer (Thiophene-Co-Pyrrole) Coating for Carbon Steel Alloy at Different Deposition Times in 3.5% NaCl Solution. Int. J. Corros. Scale Inhib..

[23] Pahuja P., Malik R., Saini N., Kumar S., Lata S. (2020). PPy–TiO_2_–Phenylalanine Composites as Anticorrosive Coatings Electrodeposited over Fe–C Steel Surface in the Marine Environment. J. Adhes. Sci. Technol..

[24] Ali M.R.R., Honeyman J.S., Domalanta M.R.B., Caldona E.B. (2024). Superhydrophobic, Highly Adhesive, and Corrosion-Protective Fluoropolymer-Modified Polythiophene Coatings. Ind. Eng. Chem. Res..

[25] Hernández-Martínez D., León-Silva U., Nicho M.E. (2015). Corrosion protection of steel by poly(3-hexylthiophene) polymer blends. Anti-Corros. Methods Mater..

[26] Branzoi F., Băran A., Mihai M.A., Paraschiv A. (2025). Anticorrosive Effect of New Polymer Composite Coatings on Carbon Steel in Aggressive Environments by Electrochemical Procedures. Coatings.

[27] Huang H., Hou L., Du H., Wei H., Liu X., Wang Q., Wei Y. (2024). In Situ Electrochemical Deposition Enables Dense Polypyrrole Grown on AZ31 Surface for Improving Corrosion Resistance. Mater. Lett..

[28] Ashassi-Sorkhabi H., Kazempour A. (2020). Incorporation of Organic/Inorganic Materials into Polypyrrole Matrix to Reinforce Its Anticorrosive Properties for the Protection of Steel Alloys: A Review. J. Mol. Liq..

[29] García-Cabezón C., Godinho V., Pérez-González C., Torres Y., Martín-Pedrosa F. (2023). Electropolymerized Polypyrrole Silver Nanocomposite Coatings on Porous Ti Substrates with Enhanced Corrosion and Antibacterial Behavior for Biomedical Applications. Mater. Today Chem..

[30] Xue S., Ma Y., Miao Y., Li W. (2020). Anti-Corrosion Performance of Conductive Copolymers of Polyaniline/Polythiophene on a Stainless-Steel Surface in Acidic Media. Int. J. Nanosci..

[31] Mirzaee M., Kianpour E., Rashidi A., Rahimi A., Pourhashem S., Duan J., Iravani D., Sirati Gohari M. (2024). Construction of a High-Performance Anti-Corrosion Epoxy Coating in the Presence of Poly (Aniline-Co-Pyrrole) Nanospheres. React. Funct. Polym..

[32] Awoyemi R.F., Almtiri M., Giri H., Scott C.N., Wipf D.O. (2024). Corrosion Protective Coatings from Poly (Heterocyclic Diphenylamine): Polyaniline Analogues. ACS Appl. Polym. Mater..

[33] González-Tejera M., García M., de la Blanca E.S., Redondo M., Raso M., Carrillo I. (2007). Electrochemical synthesis of N-methyl and 3-methyl pyrrole perchlorate doped copolymer films. Thin Solid Film..

[34] Zhou W., Wu K., Zhang K., Wang Z., Liu Z., Hu S., Fang Y., He C. (2020). Studies on Corrosion Behaviors of Q235 Steel Coated by the Polypyrrole Films Doped with different dopants. Int. J. Electrochem. Sci..

[35] Yadav P.K., Prakash O., Ray B., Maiti P. (2021). Functionalized Polythiophene for Corrosion Inhibition and Photovoltaic Application. J. Appl. Polym. Sci..

[36] Duran B., Bereket G. (2012). Cyclic Voltammetric Synthesis of Poly(*N*-methyl pyrrole) on Copper and Effects of Polymerization Parameters on Corrosion Performance. Ind. Eng. Chem. Res..

[37] Xu F., Zhang M., Cui Y., Bao D., Peng J., Gao Y., Lin D., Geng H., Zhu Y., Wang H. (2022). A Novel Polymer Composite Coating with High Thermal Conductivity and Unique Anti-Corrosion Performance. Chem. Eng. J..

[38] Xie Z.-H., Teng Y., Huang Z., Feng P., Yong Q., Jiang X., Wu L. (2025). Corrosion-Resistant and Superhydrophobic Nickel-Based Composite Coating on Magnesium Alloy. Surf. Coat. Technol..

[39] Liu G., Hou F., Wang X., Fang B. (2023). Conductive Polymer and Nanoparticle-Promoted Polymer Hybrid Coatings for Metallic Bipolar Plates in Proton Membrane Exchange Water Electrolysis: A Review. Appl. Sci..

[40] Ulaeto S.B., Ravi R.P., Udoh I.I., Mathew G.M., Rajan T.P.D. (2023). Polymer-Based Coating for Steel Protection, Highlighting Metal–Organic Framework as Functional Actives: A Review. Corros. Mater. Degrad..

[41] Zhou Y., Zhang M., Hu H., Bi C., Zhang H., Ye Y., Yan C. (2025). Research Progress of Polymer-Based Coatings for Anti-Corrosion Application: A Mini Review. J. Polym. Sci..

[42] Xu H., Zhang Y. (2019). A Review on Conducting Polymers and Nanopolymer Composite Coatings for Steel Corrosion Protection. Coatings.

[43] Yan L., Deng W., Wang N., Xue X., Hua J., Chen Z. (2022). Anti-Corrosion Reinforcements Using Coating Technologies—A Review. Polymers.

